# B-Raf inhibitor vemurafenib in combination with temozolomide and fotemustine in the killing response of malignant melanoma cells

**DOI:** 10.18632/oncotarget.2610

**Published:** 2014-10-21

**Authors:** Wynand P. Roos, Steve Quiros, Andrea Krumm, Stephanie Merz, Olivier Jérôme Switzeny, Markus Christmann, Carmen Loquai, Bernd Kaina

**Affiliations:** ^1^ Institute of Toxicology, Medical University Center, Mainz, Germany; ^2^ Department of Dermatology, Medical University Center, Mainz, Germany

**Keywords:** BRAF, Temozolomide, Fotemustine, Melanoma, Vemurafenib

## Abstract

In the treatment of metastatic melanoma, a highly therapy-refractory cancer, alkylating agents are used and, for the subgroup of *BRAF^V600E^* cancers, the B-Raf inhibitor vemurafenib. Although vemurafenib is initially beneficial, development of drug resistance occurs leading to tumor relapse, which necessitates the requirement for combined or sequential therapy with other drugs, including genotoxic alkylating agents. This leads to the question whether vemurafenib and alkylating agents act synergistically and whether chronic vemurafenib treatment alters the melanoma cell response to alkylating agents. Here we show that a) *BRAF^V600E^* melanoma cells are killed by vemurafenib, driving apoptosis, b) *BRAF^V600E^* melanoma cells are neither more resistant nor sensitive to temozolomide/fotemustine than non-mutant cells, c) combined treatment with vemurafenib plus temozolomide or fotemustine has an additive effect on cell kill, d) acquired vemurafenib resistance of *BRAF^V600E^* melanoma cells does not affect MGMT, MSH2, MSH6, PMS2 and MLH1, nor does it affect the resistance to temozolomide and fotemustine, e) metastatic melanoma biopsies obtained from patients prior to and after vemurafenib treatment did not show a change in the MGMT promoter methylation status and MGMT expression level. The data suggest that consecutive treatment with vemurafenib and alkylating drugs is a reasonable strategy for metastatic melanoma treatment.

## INTRODUCTION

Malignant melanoma is a highly therapy-refractory cancer, contributing significantly to the worldwide cancer-related mortality [1]. In the metastatic stage (stage IV) it has a dismal prognosis and treatment requires systemic therapy for disease control. Over the last 30 years different treatment modalities have been used, including immunotherapy with high-dose interleukin-2 or interferon-α and/or cytotoxic chemotherapeutics such as alkylating drugs, i.e. methylating and chloroethylating agents [2]. For methylating agents dacarbazine (DTIC) and temozolomide (TMZ) are used, which have the same therapeutic index [3]. DTIC needs metabolic activation by cytochrome P450 [4] whereas TMZ decomposes spontaneously [5] both giving rise to the DNA reactive methylating species 5-(3-methyltriazen-1-yl)imidazole-4-carboximide (MTIC). The main killing DNA lesion induced by DTIC and TMZ in tumor cells is O^6^-methylguanine (O^6^MeG) [6]. O^6^MeG needs processing by the DNA mismatch repair (MMR) proteins MSH2, MSH6, PMS2 and MLH1, which converts it during replication into DNA double-strand breaks (DSB) that trigger apoptosis [7] and senescence [8]. The damage also induces autophagy, which in glioma cells counteracts the killing response to TMZ [9].

In contrast to DTIC and TMZ, chloroethylating agents such as lomustine, nimustine, carmustine and fotemustine (FM) induce O^6^-chloroethylguanine (O^6^ClEtG) in the DNA, which is the principal critical cytotoxic DNA damage. O^6^ClEtG is unstable and is converted into a DNA interstrand crosslink (ICL) between guanine and cytosine [10]. ICLs are powerful blockers of transcription and replication, resulting in cell death. FM is used as a second line therapeutic in melanoma therapy [11], notably for the treatment of brain metastases [12, 13].

The DNA lesions O^6^MeG and O^6^ClEtG are repaired by O^6^-methylguanine-DNA methyltransferase (MGMT) in a single step reaction that inactivates MGMT [14, 15]. The amount of MGMT in the tumor is therefore a key node in alkylating drug resistance [16, 17]. Since melanomas express low amounts of MGMT [16, 17] they are expected to respond to alkylating agent based therapy, which is likely the reason why DTIC, TMZ and FM have been approved for therapy. Despite low MGMT levels in melanoma, the response rate with these genotoxic anticancer drugs remains low and the therapeutic outcome poor [18]. This could be due to silencing of downstream cell death pathways [19, 20] or due to acquired resistance as a result of increased MGMT expression or increased interstrand crosslink repair capacity [21, 22].

A breakthrough in melanoma therapy was provided by the discovery that up to 66% of malignant melanomas are mutated in *BRAF* [23]. The majority of these mutations, around 80%, lead to a change of valine to glutamic acid at codon 600, rendering the kinase constitutively active and permanently triggering the Ras-Raf-MAP kinase pathway that stimulates proliferation [23]. Specific inhibitors of mutated B-Raf have been developed which target *BRAF^V600E^* cells. One of these is vemurafenib (PLX4032) [24], which is beneficial for melanoma patients exhibiting the *BRAF*^V600E^ mutation [25]. The response rate of these patients is about 50% with significant tumor regression [25]. However, in most cases the initial phase of tumor regression is followed by therapy inefficiency and tumor progression leading finally to the death of patients [26]. The disease relapse indicates fast development of vemurafenib resistance in a subset of tumor cells that leads to their outgrowth despite continuous B-Raf inhibitor treatment.

In view of the inefficiency of genotoxic drug and B-Raf inhibitor therapy, the question arises as to combination strategies, either as concomitant or sequential treatment. *In vitro* data regarding the response of melanoma cells to TMZ or FM plus vemurafenib are not available. This prompted us to study both drugs in combination. We specifically addressed the following questions. a) Does simultaneous treatment of melanoma cells with vemurafenib and TMZ or FM provoke synergistic cell kill? b) Does chronic treatment with vemurafenib cause vemurafenib resistance *in vitro* and is this accompanied by a change in MGMT activity? c) Are vemurafenib resistant *BRAF^V600E^* melanoma cells still responsive to TMZ or FM? d) Does vemurafenib treatment change the *MGMT* promoter methylation status of melanoma tumors *in vivo*? Our data did not reveal a synergistic effect for both drugs, but encourage a sequential application as vemurafenib resistant cells did not display a change in the MGMT status and retained the killing response towards TMZ and FM.

## RESULTS

### BRAF^V600E^ is not predictive for the killing response of melanoma lines to TMZ or FM

**Figure 1:**
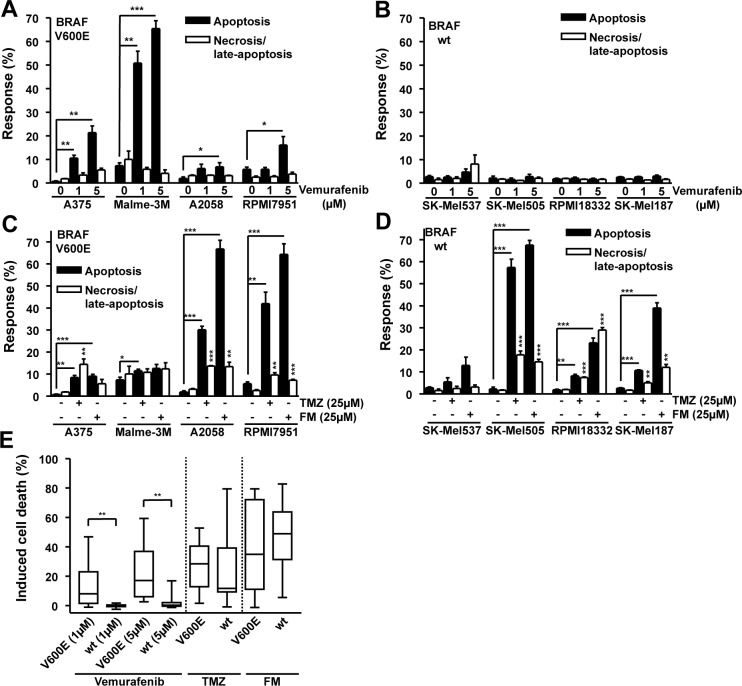
Apoptosis and necrosis/late-apoptosis induced by TMZ, FM or vemurafenib Cells were treated with the chemotherapeutic and 120h later the response was assayed. Presented data are from at least three independent experiments. *p<0.05, **p<0.005, ***p<0.0001. For all alkylating agent experiments, MGMT was depleted using the specific inhibitor O^6^BG (10 μM) by adding it to the cells 1h before TMZ or FM. Response of *BRAF* mutant (A) and wild-type (B) cells following vemurafenib addition. Response of *BRAF* mutant (C) and wild-type (D) cells following TMZ or FM addition. (E) Induced cell death, obtained by combining apoptosis and necrosis/late-apoptotic data from figures 1A, 1B, 1C and 1D, for *BRAF* mutant versus wild-type cells.

In an effort to determine whether the B-Raf inhibitor vemurafenib may have a beneficial or detrimental effect on melanoma cells treated with the genotoxic chemotherapeutics TMZ and FM, a panel of melanoma cell lines was experimentally examined. A375, Malme-3M, A2058 and RPMI7951, all containing *BRAF^V600E^* [27, 28], and SK-Mel537, SK-Mel505, RPMI18332 and SK-Mel187, wild-type for *BRAF* [29, 30], were exposed to 1 and 5 μM vemurafenib. The lines containing *BRAF^V600E^* showed a significant increase in apoptosis following vemurafenib compared to the untreated controls (Fig. 1A) while the wild-type lines did not respond to the drug (Fig. 1B). Exposing the same panel of cell lines to either 25 μM TMZ or 25 μM FM caused a different spectrum of responses, independent of *BRAF^V600E^* mutation. The methylating agent TMZ induced significant levels of apoptosis in A375, Malme-3M, A2058, RPMI7951, SK-Mel505, RPMI18332 and SK-Mel187 compared to the untreated controls (Fig. 1C and Fig. 1D). TMZ also caused significant increases in necrosis (defined by PI staining) in A375, A2058, RPMI7951, SK-Mel505, RPMI18332 and SK-Mel187 compared to the untreated controls (Fig. 1C and 1D). The chloroethylating agent FM induced significant levels of apoptosis in A375, A2058, RPMI7951, SK-Mel505, RPMI18332 and SK-Mel187 compared to the untreated controls while also causing significant increases in necrosis (PI positive) in the cell lines A2058, RPMI7951, SK-Mel505, RPMI18332 and SK-Mel187 compared to the untreated controls (Fig. 1C and 1D). Overall, the response of the lines to TMZ and FM was unrelated to *BRAF^V600E^*. For most cell lines the apoptotic response was clearly more pronounced than the necrotic response. We should also note that the level of necrosis did not parallel apoptosis, reflecting the view that separate pathways are involved. Summarizing the results, it becomes apparent that *BRAF^V600E^* predicts the response of melanoma cells to vemurafenib (tested with a concentration of 1 and 5 μM) as *BRAF^V600E^* cells were significantly more sensitive than the wild-type, while *BRAF^V600E^* did not predict the response to TMZ and FM (Fig. 1E). From these data it can be concluded that vemurafenib, TMZ and FM primarily trigger the induction of apoptotic cell death and that *BRAF*^V600E^ does not impact on the TMZ and FM killing response of melanoma cells.

### Inhibition of B-Raf (V600E) by vemurafenib does not impede or promote the genotoxic properties of TMZ or FM

**Figure 2:**
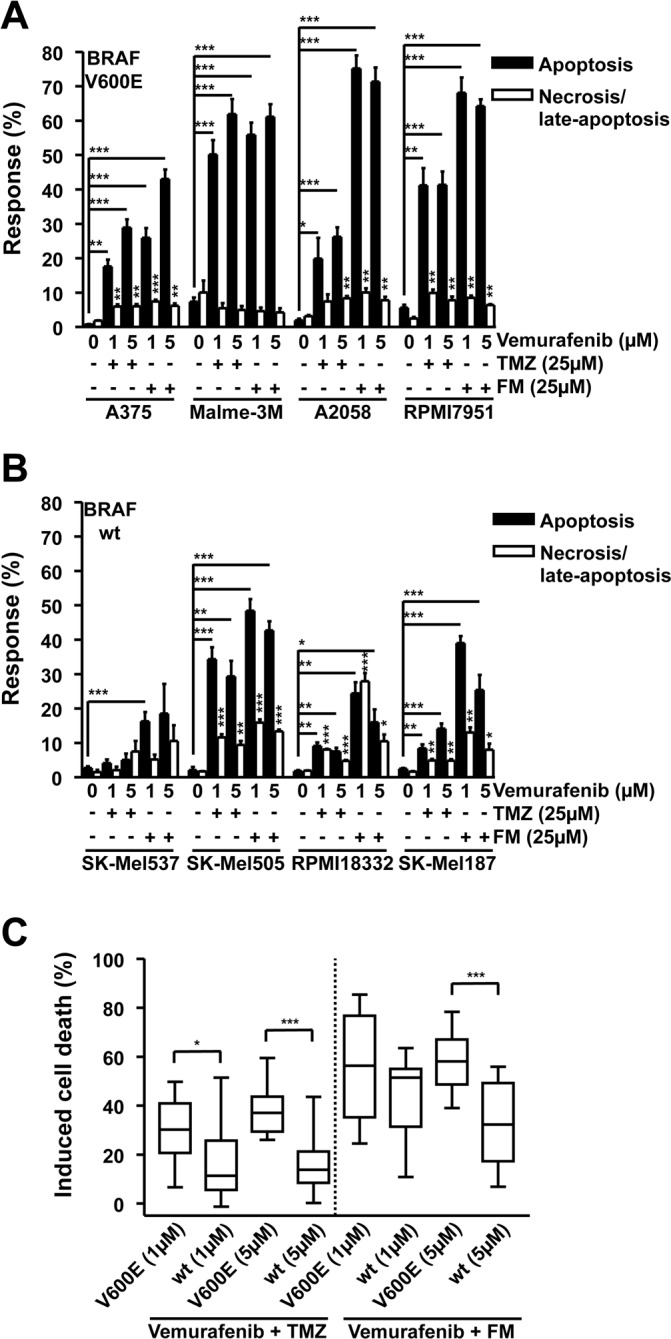
Apoptosis and necrosis/late-apoptosis induced by combination treatment with TMZ and vemurafenib or FM and vemurafenib Cells were treated with the chemotherapeutic and 120h later the response was assayed. Presented data are from at least three independent experiments. *p<0.05, **p<0.005, ***p<0.0001. MGMT was depleted with O^6^BG (10 μM) 1h before TMZ or FM. Response of *BRAF* mutant (A) and wild-type (B) cells following vemurafenib addition in combination with either TMZ or FM. Vemurafenib was added to the cells 1h after TMZ or FM. (C) Induced cell death, obtained by combining apoptosis and necrosis/late-apoptotic data from figures 2A and 2B, for *BRAF* mutant versus wild-type cells.

In order to address whether combinational treatment of melanoma cells with vemurafenib and TMZ or FM would be beneficial, the panel of melanoma cell lines was treated with 25 μM TMZ or 25 μM FM and one hour later with 1 or 5 μM vemurafenib. Vemurafenib in combination with TMZ induced apoptosis significantly in A375, Malme-3M, A2058, RPMI7951, SK-Mel505, RPMI18332, and SK-Mel187 compared to untreated controls while also significantly increasing the necrosis/late-apoptosis levels in A375, A2058, RPMI7951, SK-Mel505, RPMI18332 and SK-Mel187 compared to untreated controls (Fig. 2A and 2B). Vemurafenib in combination with FM induced significant levels of apoptosis in A375, Malme-3M, A2058, RPMI7951, SK-Mel537, SK-Mel505, RPMI18332 and SK-Mel187 compared to untreated controls while also significantly inducing necrosis/late-apoptosis in A375, A2058, RPMI7951, SK-Mel505, RPMI18332 and SK-Mel187 compared to untreated controls (Fig. 2A and 2B). Similar with what was observed with the single drug treatments, vemurafenib, TMZ and FM, the dominant cell death pathway induced in the majority of the cell lines was apoptosis. Although these data show that combining these two chemotherapeutics causes only a slight, but insignificant, increase in cell death compared to single treatments, all, except for one, responded to the combination treatment. The exception was SK-Mel537 treated with vemurafenib and TMZ. Summarizing the data, it becomes clear that alkylating agents neither prevent vemurafenib from exerting its killing effect nor does vemurafenib impact on killing induced by the alkylating agents as vemurafenib in combination with TMZ or FM showed significantly more cell kill in *BRAF^V600E^* cells than in the wild-type lines (Fig. 2C). It can be concluded that melanoma cells respond to combination treatment, irrespective of the *BRAF^V600E^* status.

### Inhibition of B-Raf (V600E) by vemurafenib does not impede or promote the proliferation inhibitory properties of TMZ or FM

**Figure 3:**
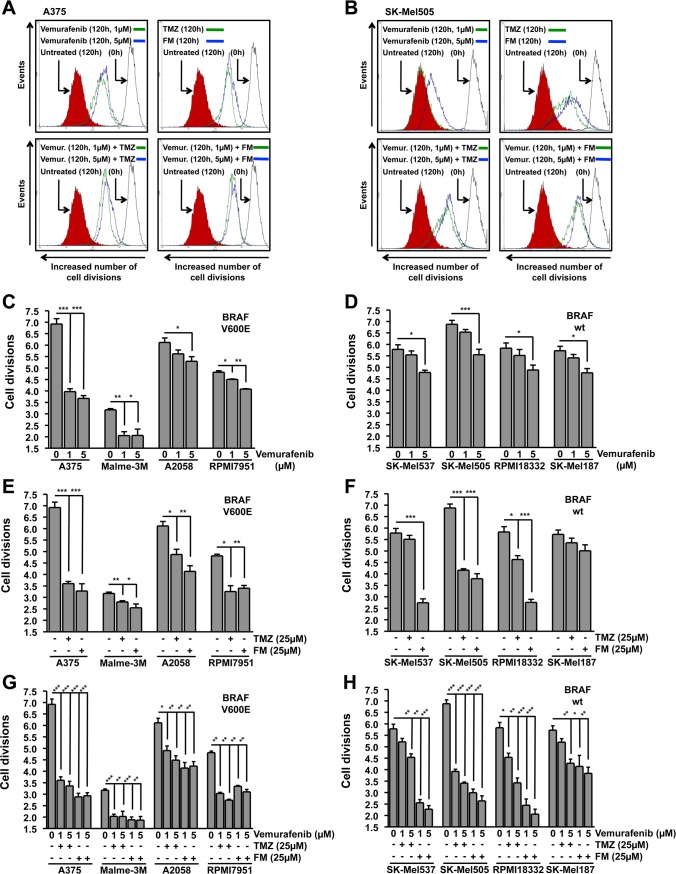
Inhibition of cell division Cells were treated with the chemotherapeutic and the number of cell divisions following 120h incubation was determined using flow cytometry of CFSE stained cells. Presented data are from at least three independent experiments. *p<0.05, **p<0.005, ***p<0.0001. MGMT was depleted with O^6^BG (10 μM) 1h before TMZ or FM. Representative flow cytometry histograms for *BRAF* mutant (A375) (A) and wild-type (SK-Mel505) (B) cells. The solid black line on the right represent CFSE stained cells at 0h, filled red histogram on the left represent untreated CFSE stained cells following 120h incubation while green and blue lines represent CFSE stained cells treated with indicated chemotherapeutics following 120h incubation. Quantification of the inhibition of cell division in *BRAF* mutant (C) and wild-type (D) cells following vemurafenib addition. Quantification of the inhibition of cell division in *BRAF* mutant (E) and wild-type (F) cells following either TMZ or FM addition. Quantification of the inhibition of cell division in *BRAF* mutant (G) and wild-type (H) cells following vemurafenib addition in combination with either TMZ or FM. Vemurafenib was added to the cells 1h after TMZ or FM.

Having determined that vemurafenib, TMZ and FM trigger cell death in melanoma cells and that combinations of vemurafenib with TMZ or FM do not impede the killing effects of the individual drugs, we expanded our examination to the influence that these treatment schedules have on the proliferation capacity of melanoma cell lines. Using the carboxyfluorescein diacetate succinimidyl ester (CFSE) proliferation assay, the cell division rate following chemotherapeutic treatment was determined. In Fig. 3A and 3B representative flow cytometry histograms are presented for A375 (*BRAF^V600E^*) and SK-Mel505 (*BRAF* wild-type) cells, respectively. Interestingly, both groups of melanoma cell lines, those containing *BRAF^V600E^* and those wild-type for *BRAF*, showed significant inhibition of cell division following vemurafenib treatment compared to controls (Fig. 3C and 3D), although the inhibitory effect was most pronounced in the *BRAF^V600E^* lines. The inhibition of proliferation observed in the wild-type cell lines (Fig. 3D) following vemurafenib was only observed at high concentration and did not lead to the induction of cell death (Fig. 1B). Treatment with either TMZ or FM caused inhibition of proliferation in the majority of the cell lines, independent of the *BRAF^V600E^* status (Fig. 3E and 3F). The exceptions being SK-Mel537 treated with TMZ and SK-Mel187 treated with both TMZ and FM. In general, FM was more effective at inhibiting cellular proliferation than TMZ at equimolar concentrations, which could be due to the ICLs induced by FM being a more effective inhibitor of proliferation. The combination treatments showed significant inhibition of proliferation in all the cell lines (Fig. 3G and 3H). Similar to the apoptosis data presented in Fig. 2, a slight increase in inhibition of proliferation was observed when applying the chemotherapeutics in combination compared to single treatment. This increase, however, was not significant. Next, we compared the lines in the combination treatment schedule. The results displayed in Fig. 3G and 3H support the conclusions drawn from the apoptosis data (Fig. 2A and 2B). When combining vemurafenib with the genotoxic drugs, a higher fraction of melanoma cell lines showed a response compared to single treatments.

### Effect of differential scheduling of treatment with vemurafenib and TMZ or FM

**Figure 4:**
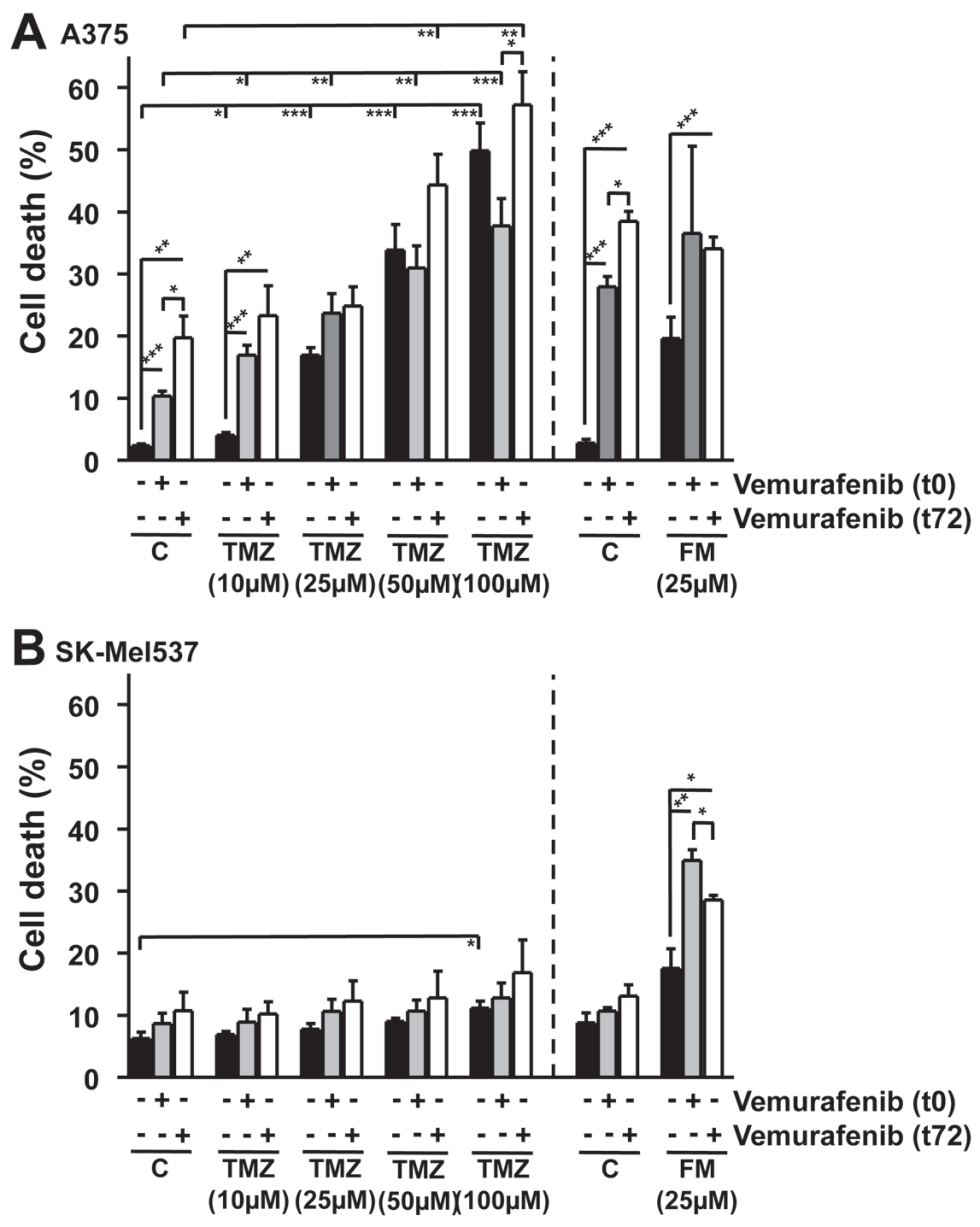
Apoptosis and necrosis/late-apoptosis induced in *BRAF* mutant and wild-type melanoma cell lines Cell death induced in *BRAF* mutant (A375) (A) and wild-type (SK-Mel537) (B) cells following vemurafenib (5 μM) addition in combination with either TMZ or FM. MGMT was depleted with O^6^BG (10 μM) 1h before TMZ or FM. Vemurafenib was added to the cells either 1h after TMZ and FM (t0) or 72h after TMZ and FM (t72) addition. Apoptosis and necrosis/late-apoptosis was determined 120 h after TMZ or FM addition. Presented values are the sum of the apoptotic and the necrotic/late-apoptotic results. Presented data are from at least three independent experiments. *p<0.05, **p<0.005, ***p<0.0001.

TMZ and FM require S-phase progression in order to exert cell death at clinically relevant concentrations [31-33]. As vemurafenib slows down proliferation (Fig. 3C and 3D), we hypothesized that adding vemurafenib simultaneously with TMZ or FM would be less effective than a sequential treatment strategy. Therefore, A375 (*BRAF^V600E^*) and SK-Mel537 (wild-type) cells were exposed to increasing concentrations of TMZ in the presence or absence of vemurafenib, added either 1h (t0) or 72h after TMZ (t72). A375 cells showed a dose dependent increase in cell death following TMZ (Fig. 4A). Co-treatment with vemurafenib and the lowest TMZ dose (10 μM), which was on its own nearly ineffective, significantly stimulated cell death. At higher dose levels this increase was not significant. There was a tendency of increased cell death in the t72 treatment schedule, when vemurafenib was added sequentially, i.e. 3 days after TMZ, which is basically in line with the supposition outlined above. There was also no significant difference between A375 cells treated with vemurafenib at t0 or t72 in combination with FM, although vemurafenib at t72 in combination with FM was more effective in eliciting cell death than vemurafenib at t0 as adding vemurafenib at t72 showed a significant increase while adding vemuravenib at t0 did not (Fig. 4A). In *BRAF* wild-type SK-Mel537 cells, TMZ was less effective, inducing significant levels of apoptosis only at the highest concentration used (100 μM) (Fig. 4B). Neither adding vemurafenib at t0 nor at t72 had any effect on TMZ-induced death. Following treatment with FM and vemurafenib, both t0 and t72 showed significant increases compared to FM alone (Fig. 4B). The results show that TMZ with vemurafenib applied simultaneously would most likely not improve the response while differential scheduling might be of some benefit for enhancing cell death following FM in combination with vemurafenib.

### Acquired resistance to vemurafenib does not influence the response of melanoma cells to TMZ or FM

**Figure 5:**
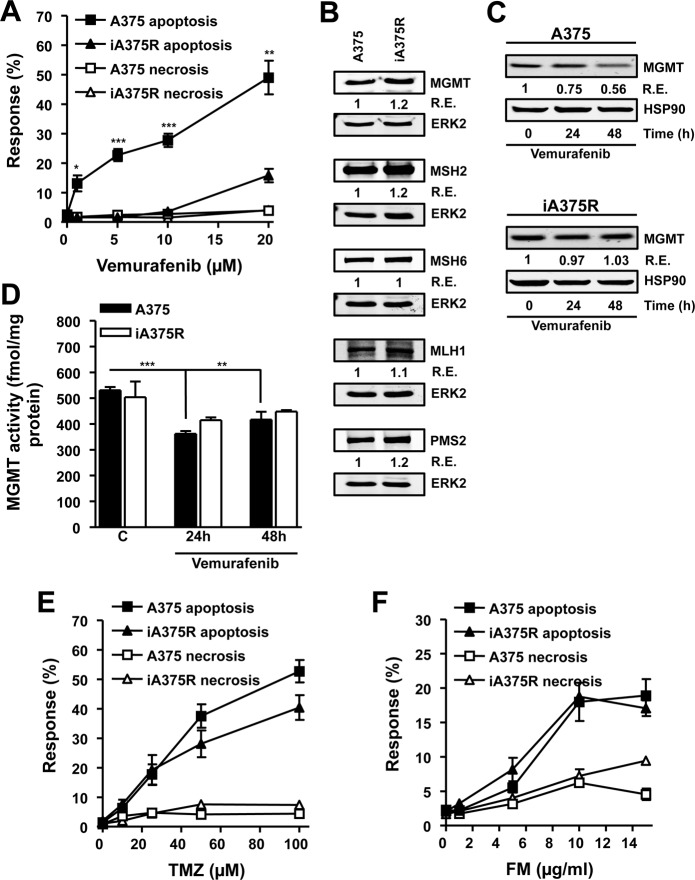
Characterization of *BRAF* mutant cells that have acquired resistance to vemurafenib (A) Response of *BRAF* mutant (A375) and vemurafenib resistant *BRAF* mutant cells (iA375R) to vemurafenib. Cells were treated with vemurafenib and 120h later the response was assayed. (B) Immunoblots of MGMT, MSH2, MSH6, PMS2 and MLH1 in A375 and iA375R cells. ERK2 served as loading control. Immunoblots of MGMT protein (C) and MGMT activity (D) in *BRAF* mutant (A375) and vemurafenib resistant *BRAF* mutant cells (iA375R). Cells were treated with vemurafenib (5 μM) and samples were harvested at indicated times. HSP90 served as loading control. (E and F) Response of A375 and iA375R to TMZ (E) and FM (F). Cells were treated with the indicated concentrations of the alkylating agents and 120h later the response was assayed. Presented data are from at least three independent experiments. *p<0.05, **p<0.005, ***p<0.0001.

With the intention of addressing whether melanoma cells that have acquired resistance to vemurafenib would exhibit cross-resistance to alkylating agents, a cell line that carries *BRAF^V600E^* (A375) was chronically exposed to vemurafenib (5 μM) for two months and the resulting resistant cell line (iA375R) was tested for cross-resistance to TMZ and FM. Vemurafenib induced significantly higher levels of apoptosis throughout the concentration range used (5-20 μM) in A375 cells compared to iA375R (Fig. 5A), demonstrating that iA375R acquired a vemurafenib resistant phenotype. Melanoma cells are protected from TMZ and FM induced apoptosis by the DNA repair protein MGMT [7], while the MMR proteins MSH2, MSH6, PMS2 and MLH1 convert the TMZ-induced O^6^MeG lesion into a cytotoxic DSB [7]. Therefore the protein levels of MGMT, MSH2, MSH6, PMS2 and MLH1 were determined in A375 and iA375R cells as any changes in these DNA repair proteins during the acquisition of resistance to vemurafenib may have an influence on cell death following TMZ. As shown in Fig. 5B, the expression of MGMT, MSH2, MSH6, MLH1 and PMS2 in the parental and vemurafenib resistant cell lines were comparable.

Since MGMT is the key factor of alkylation drug resistance, we explored its expression more thoroughly. We observed that vemurafenib caused a slight, but significant decrease in MGMT protein (Fig. 5C) and enzyme activity (Fig. 5D) in A375 cells, while iA375R cells did not show this decrease (Fig. 5C and 5D). Next, the question of whether acquired resistance to vemurafenib-induced apoptosis would lead to cross-resistance to TMZ or FM was addressed. No significant differences were observed in TMZ (Fig. 5E) or FM (Fig. 5F) induced apoptosis in A375 cells compared to iA375R cells, showing that acquired vemurafenib resistance does not lead to cross-resistance towards TMZ or FM.

### Vemurafenib therapy does not change the MGMT status of tumors *in vivo*

**Figure 6:**
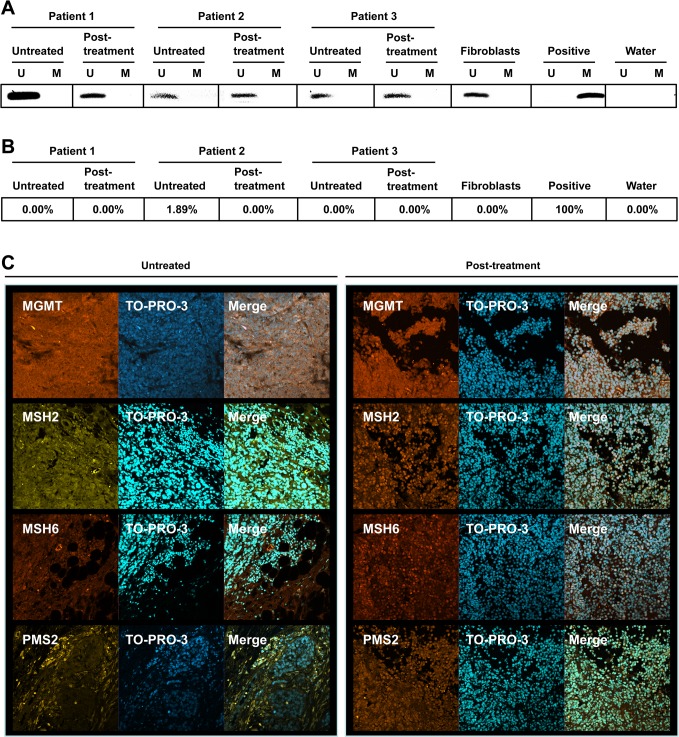
MGMT, MSH2, MSH6 and PMS2 status of melanomas MSP (A) and MS-HRM (B) of the *MGMT* promoter in paired tumors from three patients before and after vemurafenib treatment. U depicts an unmethylated promoter while M depicts a methylated promoter. Fibroblasts served as negative control (unmethylated promoter) and LN229 cells served as positive control (methylated promoter). (C) Microphotographs of paired tumors before and after vemurafenib. MGMT, MSH2, MSH6 and PMS2 protein was detected using immunohistochemistry. Nuclei were stained with TO-PRO-3.

As MGMT plays such an important role in the resistance of cells and tumors to alkylating agents and a small, but significant, change in MGMT activity was observed following *in vitro* treatment of *BRAF^V600E^* cells with vemurafenib (Fig. 5D), the promoter methylation status of the *MGMT* gene was determined in paired samples obtained from patients before and after vemurafenib therapy. Fibroblasts were used as a negative control and the glioma cell line LN229 that contains a methylated promoter served as positive control. These data revealed that tumors *in situ* displayed unmethylated *MGMT* before and after vemurafenib treatment (Fig. 6A), indicating that vemurafenib therapy had no impact on the *MGMT* promoter methylation status of melanoma.

To support the MSP data, the *MGMT* promoter methylation status of these tumors was also determined by MS-HRM analysis. Similar to what was found for the MSP assay, the methylation status of the *MGMT* promoter did not change during therapy with vemurafenib as the pre-treatment tumors and the post-treatment tumors all showed unmethylated promoters (Fig. 6B). Next, the MGMT protein expression was determined in untreated and vemurafenib treated tumors using IHC. Both the untreated and the vemurafenib treated tumors stained positive for MGMT (Fig. 6C). Interestingly, the post-treatment tumors showed more staining for nuclear localized MMR proteins MSH2, MSH6 and PMS2 than the untreated tumor (Fig. 6C), which can be taken to indicate enhancement of MMR capacity of vemurafenib treated tumors. Collectively, regarding MGMT, it can be concluded from the data that vemurafenib does not cause a change in the *MGMT* promoter methylation status.

## DISCUSSION

The gold standard in the chemotherapy of metastatic malignant melanoma is DTIC or TMZ. Despite this genotoxic therapy, the disease has a dismal prognosis. The finding that 40 to 60% of malignant melanomas are mutated in *BRAF* [23, 34] paved the way for searching for specific small molecule inhibitors. One of these is vemurafenib [24], which causes significant tumor regression in metastasized *BRAF*^V600E^ mutated melanoma patients [25]. Since tumor regression is only transient in most cases, followed by acquired drug resistance and tumor progression [26], the search for alternative therapeutic strategies is warranted. It would be reasonable to consider the idea of combining vemurafenib with the classical anticancer drugs such as TMZ, a representative methylating agent, and FM, a representative chloroethylating nitrosourea, applied for melanoma treatment [35]. Therefore, in this study, we addressed how vemurafenib influences the response of melanoma to alkylating agents.

The data show that *BRAF^V600E^* sensitizes melanoma cells to vemurafenib-triggered apoptosis compared to wild-type cells. This is most likely due to the phenomenon of oncogene addiction [36]: as soon as mutant B-Raf (V600E) is inhibited cells initiate apoptosis. Contrary to the results obtained with vemurafenib, no systematic and significant differences were observed in the apoptosis levels triggered by TMZ or FM in *BRAF^V600E^* versus wild-type cells, showing that *BRAF^V600E^* status does not influence the response of melanomas to alkylating agents. We should note that at the used concentration of TMZ and FM, the clinical relevant DNA lesions O^6^MeG and O^6^ClEtG are responsible for triggering apoptosis in melanoma cells [7]. For this reason we performed all experiments by inhibiting the repair enzyme MGMT prior to TMZ or FM treatment, having a firm basis for comparing the O^6^-alkylguanine response in the cell lines. The concentration of O^6^BG added was sufficient to inactivate MGMT for the duration of the experiments. Combining vemurafenib with TMZ or FM did not clearly reduce the killing properties of the chemotherapeutics. This is a pivotal finding because it shows that not only does vemurafenib and the alkylating agents exert their killing effects via independent pathways, but that there is no mechanistic reason why these therapeutics should not be combined during therapy. This may be of benefit in a heterogeneous tumor, or in the 19% of patients that carry both *BRAF^V600E^* and wild-type tumors [37], where combination therapy could lead to better control. Of interest, vemurafenib caused a slowdown in replication rate in both *BRAF^V600E^* and wild-type cells. In wild-type, however, this only occurred at higher concentrations, which is consistent with the specificity of the B-Raf inhibitor [24]. Alkylating agents in combination with vemurafenib lead to an even more pronounced slowdown in replication rate, lending support for the use of combinational therapy.

Addressing the question of cross-resistance, we generated vemurafenib resistant *BRAF^V600E^* cells by chronic exposure of melanoma cells to the serine/threonine-protein kinase B-Raf inhibitor vemurafenib. These cells did not show a change in the key resistant marker MGMT [14] on protein or activity level. Therefore, we conclude that chronic vemurafenib treatment has no impact on MGMT, which is supported by our data obtained with tumor specimens. Interestingly, treating the *BRAF^V600E^* cell line with a single dose of vemurafenib, a transient down-regulation of MGMT on protein and activity level was observed. This might be a result of transient growth changes that impact MGMT promoter activity. Cells, however, still expressed MGMT. Under these conditions N-alkylation lesions trigger cell death, which require high dose treatment with alkylating agents that are presumably clinically not relevant. As outlined above, in this experimental setting the response of melanoma cells depleted in MGMT was assessed in order to elucidate the influence of vemurafenib on the O^6^-alkylguanine response. To this end, we inhibited MGMT by O^6^BG. Although MGMT inhibitors are not routinely applied in melanoma therapy, the conclusions can be translated to the therapeutic situation as TMZ/DTIC is being used daily, which is supposed to cause a depletion of MGMT in tumor cells and MGMT in melanoma has been shown to be predictive of outcome [38].

We show that once MGMT was inhibited the parental and vemurafenib resistant cells displayed a similar killing response following treatment with FM and TMZ. This indicates that there was no cross-resistance between vemurafenib blocking the B-Raf pathway and O^6^MeG and O^6^ClEtG triggered signaling leading to cell death following TMZ and FM, respectively. The above discussed results suggest that combinational treatment of melanoma with vemurafenib and alkylating agents may be beneficial and that switching to alkylating agent based therapy once tumors has acquired resistance to vemurafenib is feasible and might be of therapeutic benefit.

The expression of *MGMT* in melanomas is controlled by the methylation status of CpG islands in its promoter [38]. Therefore, the influence of vemurafenib therapy on the silencing of *MGMT* in melanomas was investigated by determining the promoter methylation status in tumor specimens. Here we show that vemurafenib did not alter *MGMT* promoter methylation as pre-vemurafenib and post-vemurafenib treated cancers all contained unmethylated promoters, showing that vemurafenib is not active in suppressing the transcriptional expression of *MGMT*. This was confirmed by IHC; no discernable differences in the MGMT protein levels in pre- and post-treatment tumors were observed. Interestingly, post-treatment tumors showed more pronounced nuclear staining for the MMR proteins MSH2, MSH6 and PMS2. MMR is required for processing the TMZ-induced O^6^MeG DNA lesion into a killing lesion, namely DSBs. Increased levels of these proteins in post-treatment tumors may therefore imply that following vemurafenib switching to TMZ therapy may even be beneficial.

Collectively, we report that the alkylating agents TMZ and FM do not act synergistically with vemurafenib in melanoma cells. Combination treatment with TMZ or FM with vemurafenib did not attenuate the cell killing properties of the individual chemotherapeutics, rather additivity was observed. Further, acquired resistance to vemurafenib of melanoma cells does not lead to cross-resistance to TMZ and FM. Vemurafenib caused a slight decrease in MGMT protein and activity in pulse-treated cells *in vitro* while not influencing the *MGMT* promoter methylation status in tumors following therapy. The lack of cross-resistance, along with the increased MMR protein expression observed in melanomas *in situ*, lend support for the concept of switching to TMZ, dacarbazine or FM once tumors acquire resistance to vemurafenib.

## MATERIALS AND METHODS

### Cell lines and cell culture

The *BRAF^V600E^* A375, Malme-3M, A2058 and RPMI7951 [27, 28] and wild-type SK-Mel537, SK-Mel505, RPMI18332 and SK-Mel187 [29, 30] melanoma cell lines were used in this study. A375, Malme-3M, A2058, RPMI7951 and SK-Mel187 were cultivated in DMEM while SK-Mel537, SK-Mel505 and RPMI18332 were cultivated in RPMI-1640. For Malme-3M the medium was supplemented with 20% fetal calf serum (FCS) while 10% FCS was used for the rest. In all cases, 100U/mL penicillin and 100mg/mL streptomycin were present and cells were cultivated at 5% CO_2_, 37^o^C in a humidified atmosphere. All cell lines were verified mycoplasma negative before experimental use. A375 and Malme-3M were obtained from the American Type Culture Collection, RPMI7951 from the German Cell Culture Depository while A2058, SK-Mel537, SK-Mel505, RPMI18332 and SK-Mel187 were a generous gift from Dr. William K. Kaufmann (Dept. of Pathology & Laboratory Medicine, University of North Carolina at Chapel Hill, Chapel Hill, NC, USA). All the lines were carefully characterized in the laboratory they originated from, displayed the expected phenotype, but were not reauthenticated in our laboratory.

### Drugs and drug treatment

Vemurafenib (PLX4032, Selleckchem, Absource Diagnostics GmbH, Munich, Germany) was dissolved in DMSO to a final stock concentration of 10 mM. Temozolomide (TMZ, Schering-Plough, Kenilworth, NJ, USA) stock solutions with a final concentration of 35 mM were prepared by dissolving the drug in DMSO and then diluting it in an additional two parts dH_2_O. Vemurafenib and TMZ stocks were stored at −80°C. Fotemustine (FM, Muphoran, Servier Research International, Neuilly sur Seine, France) was prepared fresh for each treatment at a stock concentration of 10 mg/ml in EtOH. O^6^-benzylguanine (O^6^BG) stock was prepared by dissolving it in DMSO to a final concentration of 10 mM. O^6^BG was always added to the cells 1h before TMZ or FM treatment to deplete MGMT, unless stated otherwise. For the combination treatments, O^6^BG was added to the medium, 1h later TMZ or FM was added and then an additional hour later (t0), or 72h later (t72), vemurafenib was added. For all apoptosis and growth inhibition experiments, samples were harvested 120 h after TMZ of FM addition and assayed for the response.

### Measurement of apoptosis by flow cytometry

Annexin V/propidium iodide double-staining of unfixed cells was used to distinguish between early apoptotic cells and late-apoptotic/necrotic cells as described [19]. Annexin V positive cells were classified as apoptotic while double-positive cells were classified as necrotic/late-apoptotic. The flow cytometric analysis was carried out using a FACS Canto II flow cytometer (Becton Dickinson GmbH, Heidelberg, Germany). The data were analyzed using the BD FACSDiva software.

### Cellular proliferation assay

The cellular division rate was determined using carboxyfluorescein diacetate succinimidyl ester (CFSE) stained cells [39]. One day after labeling the control sample (0h) was harvested and analyzed by flow cytometry (FACS Canto II). On this day cells were treated or not with vemurafenib, TMZ, FM or combinations of the drugs, and 120h later samples were harvested and subjected to flow cytometry analysis. Using the mean 0h fluorescence signal, a standard curve was plotted to determine the number of cell divisions after 120h incubation in treated and untreated cells.

### Preparation of protein extracts

Whole cell protein extracts were prepared as described [7]. Protein concentrations were determined using the Bradford method [40].

### Immunoblotting

Western blot analysis was performed as described [7]. Proteins were detected by the Odyssey 9120 Infrared Imaging System (Li-Cor Biosciences, Lincoln, Nebraska, USA). The antibodies used were anti-MGMT (Merck Millipore, Billerica, Massachusetts, USA), anti-HSP90, anti-beta-actin (Santa Cruz Biotechnology, Heidelberg, Germany), anti-MSH2 (Calbiochem, San Diego, CA, USA), anti-MSH6 (Transduction Laboratories, Lexington, KY, USA) and anti-MLH1 (BD Pharmingen, Heidelberg, Germany).

### Determination of MGMT activity

MGMT activity assay was performed as described [41]. HeLa S3 cells expressing MGMT (588±86 fmol/mg protein) and HeLa MR cells deficient in MGMT served as positive and negative controls. Data are expressed as fmol radioactivity transferred from ^3^H-labelled DNA to protein/mg of protein within the sample.

### Preparation of genomic DNA and methylation-specific PCR (MSP)

Paraffinized tumor samples were cut into 10 μm thick slices and immobilized on glass slides. One of the specimens (with 3 μm thickness) was hematoxylin stained, evaluated and the tumor area was labeled. The tumor tissue was carefully removed from 3 slides (with 10 μM thickness), genomic DNA was extracted by the standard protocol using phenol-chloroform and DNA was modified using the EZ DNA Methylation Kit from Zymo Research (Freiburg, Germany). Methylation-specific PCR (MSP) for the promoter of *MGMT* was performed as described [42]. The following primer sequences were used (5′-3′): Meth-up TTT CGA CGT TCG TAG GTT TTC GC, Meth-low GCA CTC TTC CGA AAA CGA AAC G, Unmeth-up TTT GTG TTT TGA TGT TTG TAG GTT TTT GT and Unmeth-low AAC TCC ACA CTC TTC CAA AAA CAA AAC A [43].

### Methylation specific high resolution melting curve (MS-HRM) analysis

The relative amount of CpG sites methylated in the MGMT promoter was determined by MS-HRM. Fully methylated and unmethylated DNA for the methylation standard was prepared from a Buccal swab of a healthy donor as described [44]. Tumor DNA, obtained as described in MSP section, and methylation standard DNA were bisulfite modified using the EZ DNA Methylation Kit from Zymo Research (Freiburg, Germany). Methylation independent primers for the *MGMT* promoter was used that included no CpG sites. Primers were designed using the Pyromark assay Designer 2.0 (Qiagen) that flank the binding sites of the MSP primers [43]. The following primer sequences were used (5′-3′): Up GGA TAT GTT GGG ATA GTT and low CCC AAA CAC TCA CCA AAT. Following PCR amplification and melting point analysis, performed by stepwise increase of temperature by 0.2 ^o^C, the normalized Precision Melt Analysis Software (BioRad) output were exported to GraphPad Prism. The area under the curve was calculated for all the samples, DNA standard and tumor, and the quadratic least square regression was used to interpolate the unknown samples to the standards. R^2^ was >0.97 (for linear regression R^2^>0.93).

### Tumor biopsies and immunohistochemistry

Samples of malignant melanoma were obtained from patients following surgery. Patient material was obtained with informed consent and approval from the institutional ethics committee of the University Medical Center Mainz. Slices were obtained from paraffinised sections, which were labeled as to the tumor area. Immunohistochemical analysis of MGMT, MSH2, MSH6 and PMS2 levels in untreated and post-treatment melanoma samples were performed as described [45]. Antibodies used were anti-MGMT (clone MT3.1), anti-MSH2 (clone FE11, Merck Millipore, Billerica, Massachusetts, USA), anti-MSH6 ([44]) and anti-PMS2 ([EPR3947], Abcam Inc., Cambridge, Massachusetts, USA). Following incubation with the appropriate 2^nd^ anti-body nuclei were stained with TO-PRO-3. Microphotographs were acquired by laser scanning microscopy (LSM710, Carl Zeiss MicroImaging).

### Statistics

The computer-based program GraphPad Prism version 3 was used to perform the statistical analysis. For comparing differences between two populations the unpaired t-test was used.
